# Recruitment strategies in a diverse Parkinson’s disease cohort: lessons from the East London Parkinson’s Disease Project

**DOI:** 10.1136/bmjno-2026-001732

**Published:** 2026-07-01

**Authors:** Kamalesh C Dey, Essa Bhadra, Alexandra Zirra, Ellen Camboe, Brook Huxford, Rhiannon Laban, Viktoria Azoidou, David A Gallagher, Thomas Boyle, Caroline Budu, Shafaq Hussain-Ali, Charles R Marshall, Laura J Smith, Alastair J Noyce

**Affiliations:** 1 Centre for Preventive Neurology, Wolfson Institute of Population Health, Queen Mary University of London, London, UK; 2 Department of Neurology, Barts Health NHS Trust Royal London Hospital, London, UK; 3 Centre for Psychiatry and Mental Health, Wolfson Institute of Population Health, Queen Mary University of London, London, England, UK

**Keywords:** PARKINSON'S DISEASE, CLINICAL NEUROLOGY

## Introduction

 Parkinson’s disease (PD) research has historically been under-represented in diverse populations, limiting generalisability and contributing to inequalities in healthcare access.^1^,^4^ Structural and cultural barriers, including language, health literacy, mistrust of research and practical constraints, continue to reduce engagement among diverse communities.^5 6^ This is particularly important as emerging evidence suggests that prevalence, symptom presentation and treatment experiences of PD may vary across ethnic groups.^7 8^ However, these differences are likely shaped by a complex interplay of biological, social-lifestyle, psychological, environmental and healthcare-related factors, which remain poorly characterised due to limited representative participation.

Efforts to improve engagement in diverse populations have largely been reported in the USA^9^,^11^ but are lacking for the UK context. Around 166 000 people with PD live in the UK, including approximately 14 220 individuals in London.^12^ However, ethnicity-specific prevalence data are limited due to inconsistent recording in primary care records.^6^ The East London Parkinson’s Disease (ELPD) project was established in October 2018 in East London to address these gaps in one of the most diverse and socioeconomically deprived regions in the UK.^13^ Around 50% of patients in East London are from diverse ethnic backgrounds, including South Asian (39%) and Black (11%) communities.^13^ The borough of Tower Hamlets has substantial ethnic diversity and high levels of socioeconomic deprivation,^14^ highlighting the need for tailored recruitment approaches.

The project aimed to recruit people with PD and healthy controls (HC) while embedding strategies to improve engagement among under-represented populations. Here, we outline the recruitment framework developed within ELPD and highlight lessons for future neurodegenerative disease research seeking to improve inclusion among diverse communities.

## East London Parkinson’s disease project

**Figure 1:**
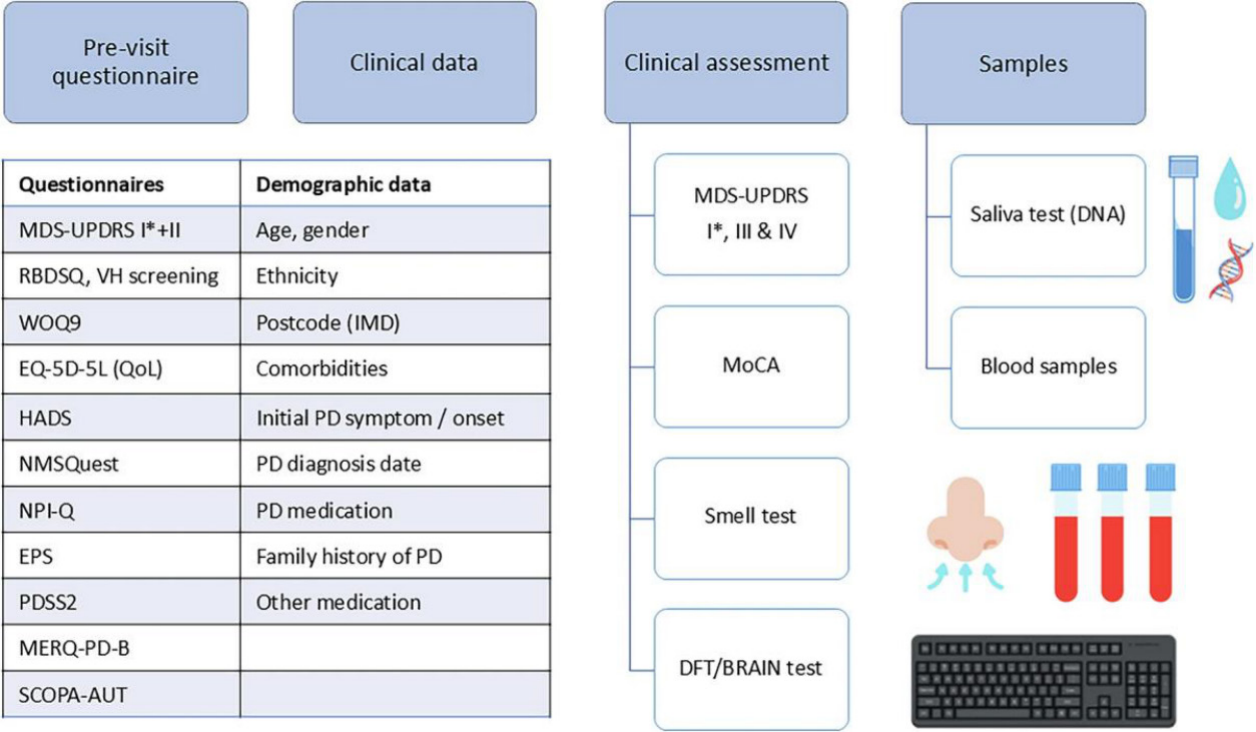
East London Parkinson’s Disease Project protocol involved one mandatory clinical visit, along with the previsit questionnaires (typically takes 3.5 hours). DFT, Distal Finger Tapping; EPS, Epworth Sleepiness Questionnaire; EQ-5D-5L QoL, EuroQol Five-Dimension Five-Level Quality of Life Questionnaire; IMD, Index of Multiple Deprivation; HADS, Hospital Anxiety and Depression Questionnaire; MDS-UPDRS, MDS-Unified Parkinson’s Disease Rating Scale; MERQ-PD-B, Mini-Environmental Risk Factor Questionnaire in Parkinson’s Disease version B; MoCA, Montreal Cognitive Assessment; NMSQ, Non-Motor Symptoms Questionnaire; PD, Parkinson’s disease; PDSS2, Parkinson’s Disease Sleep Scale 2; RBDSQ, REM Sleep Disease Screening Questionnaire; SCOPA-AUT, Scales for Outcomes in Parkinson’s Disease-Autonomic Dysfunction; VH, visual hallucinations; WOQ9, Wearing OFF Questionnaire.^13^

The ELPD is a cross-sectional, case-control study designed to characterise the clinical manifestations and determinants of PD in a diverse population in East London (protocol^13^ presented in figure 1).

People with PD were recruited from Royal London Hospital (RLH), East London and HC were recruited through the spouses of patients and community engagement activities across East London. All participants provided written informed consent prior to participation.

## Recruitment strategy framework

**Figure 2:**
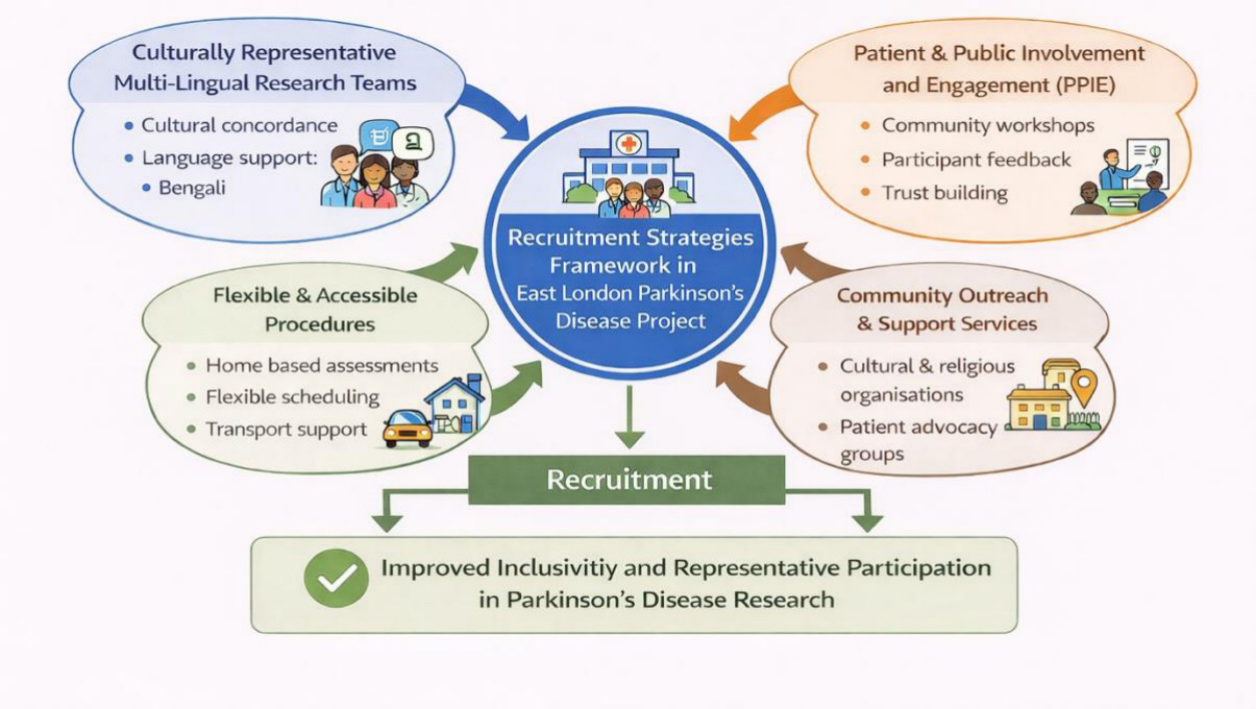
Recruitment strategies framework in the East London Parkinson’s Disease project.

A structured recruitment framework (figure 2) was developed to address structural, cultural and practical barriers affecting the engagement of under-represented populations, including South Asian, Black and other ethnic groups in East London. The framework included four integrated components: culturally representative research teams, flexible study procedures, patient and public involvement and engagement (PPIE) and community outreach.

## Recruitment strategies

### Culturally representative multilingual research team

A culturally representative and multilingual research team formed the foundation of the recruitment strategy. Researchers fluent in Bengali, Hindi, Urdu and Romanian languages were embedded within the ELPD team. This improved communication, supported informed consent and reduced language-related barriers during recruitment and assessments.

Cultural familiarity enhanced trust and helped address historical under-representation in research. Continuity of staff allowed participants to engage with familiar team members over time, strengthening rapport and likely to improve retention. Participants reported increased comfort and confidence due to consistent interactions, highlighting the importance of representation within research teams as a mechanism for improving inclusivity and trust among ELPD participants, especially among diverse groups.

### Flexible and accessible study assessments and procedures

Flexible study procedures were essential for improving participation. Home visits and clinic appointments were offered at convenient times, including evenings, to accommodate work and caregiving responsibilities. ELPD study documents, including consent forms and participant information sheets, were translated into Bengali and other relevant languages. Transport support, including taxi booking and meeting costs for travel, reduced logistical barriers where possible and study assessments aligned with clinical appointments at RLH. Participants could communicate through preferred channels, such as phone, text or email, and nominate family members for communication where appropriate.

Regular engagement included reminders, updates on study progress and newsletters distributed twice yearly through PPIE channels and community events. These measures reduced participation burden and improved inclusivity for individuals with mobility limitations, socioeconomic challenges or caregiving responsibilities.

### Patient and public involvement and engagement

PPIE was embedded as a core component of the ELPD project. A dedicated PPIE research fellow (SHA) contributed to all stages of research from design to dissemination. Community-based events brought together patients, families, healthcare professionals, funders (including Parkinson’s UK and Cure Parkinson’s) and researchers. Interactive approaches included focus groups, panel discussions, priority-setting exercises using stickers and tokens, World Café discussions and scenario testing of consent and recruitment processes.

PPIE input shaped research priorities such as non-pharmacological therapies, mental health and access to trusted information. It also improved study materials and recruitment tools with translation versions, including newsletters and the development of an informational video on lumbar puncture procedures. Regular feedback through PPIE ensured transparency and demonstrated how participant input influenced study design (‘You said, we did’). Adjustments were made to accommodate cultural and religious needs, such as modifying event arrangements during Ramadan and providing a prayer room and halal options. This strengthened trust and reinforced culturally responsive engagement in research.

### Community outreach and support services

Community outreach played a key role in recruitment. Partnerships were established with local organisations, including social, religious and voluntary organisations. These partnerships enabled engagement with individuals who may not otherwise access hospital-based research. Activities included awareness sessions, community meetings and collaboration with local support services. Outreach also involved signposting participants to relevant support networks, including PD advocacy and caregiving services, helping to address broader social and practical needs. These efforts improved awareness of PD research and strengthened trust within diverse communities.

## Impact of recruitment strategy framework

Between January 2019 and March 2025, 310 people with PD and 133 HC were recruited. Participant demographics reflected East London’s diversity, with 50% of participants with PD and 62% of HC identifying as South Asian or Black communities (online supplemental figure 1). Additional representation included Arabic, Chinese and other ethnic groups. Approximately 45%–50% of participants were from the most deprived socioeconomic quintiles (Q1 and Q2), based on the Index of Multiple Deprivation scores^15^ (table 1).

**Table 1:** Demographic characteristics

Characteristic	Patients with Parkinson’s disease (n=310)	Healthy controls (n=133)
Age at assessment (mean±SD)	68.6±11.0	62.1±13.6
Ethnicity (n, %)		
White	133 (43.9)	47 (35.3)
South Asian	120 (39.4)	74 (55.6)
Black	30 (10.3)	8 (6.0)
Other	27 (6.5)	4 (3.0)
Male (n, %)	184 (59.4)	75 (56.4)
Index of Multiple Deprivation quintile (median±IQR)	2±2	2±2

n, number of participants.

**Figure 3:**
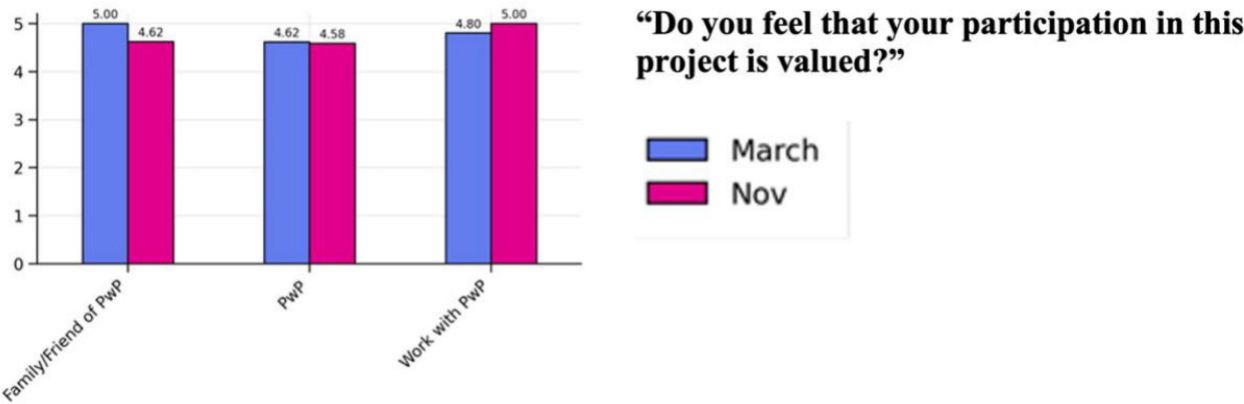
Patient and public involvement and engagement (PPIE) feedback captured across two events in 2025. Attendees, including PwP, people supporting someone with Parkinson's disease and people working with PwP (n=70) were asked. ‘Do you feel that your participation in this project is valued?’ 1=not at all, 5=very much. PwP, people with Parkinson's disease

PPIE feedback indicated participants were engaged and felt their participation in ELPD was valued (figure 3 and online supplemental figure 2). Participants particularly valued flexible procedures, multilingual support and consistent communication. These strategies improved inclusivity for individuals with language barriers, mobility limitations or competing responsibilities.

## Discussion

The ELPD demonstrates that culturally informed and community-embedded approaches can improve engagement and representation in PD research. Integrating these strategies addressed longstanding barriers such as language differences, mistrust of research and logistical challenges that have contributed to the under-representation of diverse populations.^10 11^

Improving diversity in research is essential to ensure that findings reflect the populations they aim to investigate and to better understand how social, environmental and healthcare factors shape disease presentation and outcomes. Inclusive cohorts enable this broader perspective while avoiding overinterpretation of ethnicity as a proxy for biological difference.^8^

Recruitment within ELPD closely reflects the demographics of East London, with substantial inclusion of South Asian and Black participants and individuals from socioeconomically deprived backgrounds.^13^ Representation from these communities exceeded levels typically observed in the UK-based PD studies, enhancing the generalisability of findings.^16 17^ Although recruitment was confined to a single metropolitan area and the independent contribution of each strategy was not formally quantified, the framework provides a practical and replicable model for inclusive research. Future studies should assess the scalability, cost-effectiveness and impact on longitudinal retention across different settings

Implementation of such inclusive strategies requires additional resources, including dedicated staffing, translation services, transport support and PPIE activities. In ELPD, recruitment costs were estimated to be up to four times higher for under-represented groups due to increased researcher time, flexible scheduling, translation needs and travel. These additional demands should be accounted for in study design and funding applications. At a policy level, institutions and funders must recognise the importance of investing in inclusive research infrastructure to ensure equitable access to participation. Such investment may also facilitate improved recruitment into future clinical trials in under-represented populations.^18^

Overall, culturally sensitive and community-informed strategies can effectively overcome barriers related to language, mobility and mistrust, demonstrating that research protocols tailored to practical and cultural needs can improve inclusivity and representativeness in PD research.^19 20^ Similar approaches could be applied as a model in other neurodegenerative disease studies to improve inclusivity, generalisability, engagement and meaningful participation among under-represented populations.

Strengths of the study include the real-world implementation within a highly diverse population and integration of PPIE throughout the research process. Limitations include the cross-sectional design and the absence of follow-up data using standard recruitment approaches. Further research is needed to quantify the relative impact of individual strategies within longitudinal designs to evaluate cost-effectiveness and assess generalisability across other geographic regions and healthcare settings.

## Conclusions

Culturally informed recruitment strategies successfully supported the inclusion of diverse populations in the ELPD. The integration of multilingual teams, flexible procedures, PPIE, transport support and community outreach strengthened trust, engagement and participation. These findings provide a practical model for future neurodegenerative disease research aiming to improve equity, inclusivity and generalisability across diverse populations.

## Supplementary material

10.1136/bmjno-2026-001732 online supplemental file 1
