# Supplementary material for: Recruitment strategies in a diverse Parkinson’s disease cohort: lessons from the East London Parkinson’s Disease Project
Source: BMJ Neurol Open. 2026 Jul 1;8(2):e001732. doi: 10.1136/bmjno-2026-001732 (PMC13330958; doi:10.1136/bmjno-2026-001732)
Supplement: online supplemental file 1 [file bmjno-8-2-s001.pdf]

Supplemental Figure:

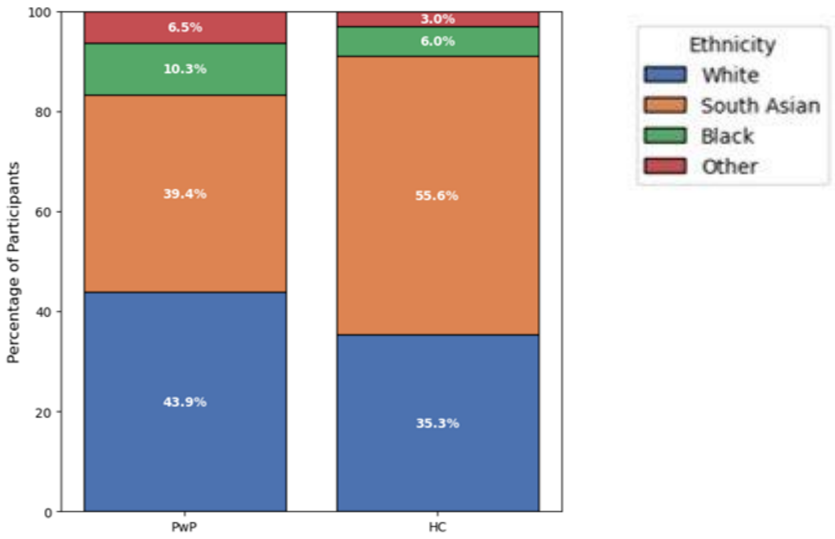

Supplemental Figure 1. Ethnicity distribution in patient with Parkinson's disease (PwP) and healthy controls (HC) in East London Parkinson's Disease (ELPD) Project. Around 56% of patients and 64% of controls were from underrepresented population groups, including South Asian or Black or other.

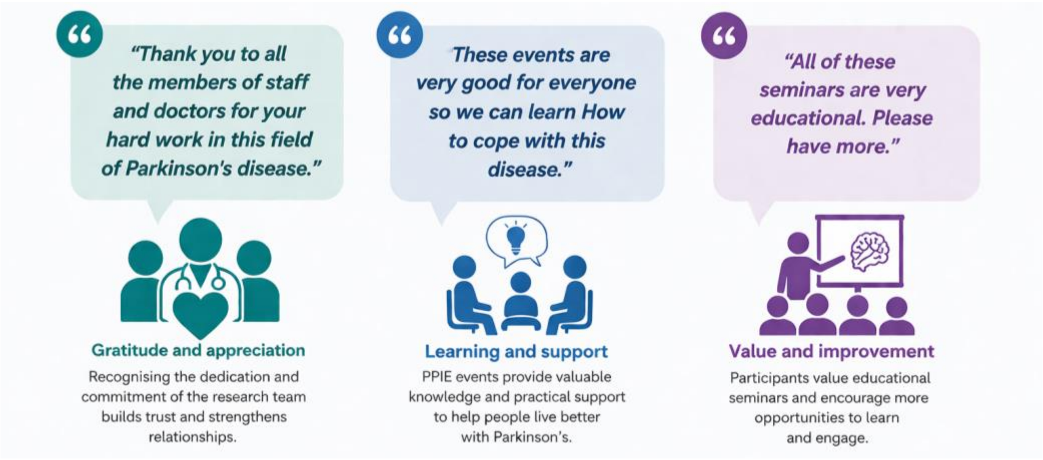

Supplemental Figure 2. Illustrative quotes highlighting positive experiences of Patient and Public Involvement and Engagement (PPIE) events in 2025 are provided in the panel below.
